# Temporomandibular joint regeneration: proposal of a novel treatment for condylar resorption after orthognathic surgery using transplantation of autologous nasal septum chondrocytes, and the first human case report

**DOI:** 10.1186/s13287-018-0806-4

**Published:** 2018-04-07

**Authors:** Ricardo de Souza Tesch, Esther Rieko Takamori, Karla Menezes, Rosana Bizon Vieira Carias, Cláudio Leonardo Milione Dutra, Marcelo de Freitas Aguiar, Tânia Salgado de Sousa Torraca, Alexandra Cristina Senegaglia, Cármen Lúcia Kuniyoshi Rebelatto, Debora Regina Daga, Paulo Roberto Slud Brofman, Radovan Borojevic

**Affiliations:** 1 Centro de Medicina Regenerativa, Faculdade de Medicina de Petrópolis – FASE, Avenida Barão do Rio Branco 1003, Centro, Petrópolis, RJ 25680-120 Brazil; 2 Instituto de Saúde de Nova Friburgo, Universidade Federal Fluminense, Rua Dr. Silvio Henrique Braune 22, Nova Friburgo, RJ 28625-650 Brazil; 3 Hospital Universitário Clementino Fraga Filho, Universidade Federal do Rio de Janeiro, Avenida Pedro Calmon, 550 – Cidade Universitária, Rio de Janeiro, RJ 21941-901 Brazil; 4 Centro de Tecnologia Celular, Pontifícia Universidade Católica do Paraná, Rua Imaculada Conceição 1155, Bairro Prado Velho, Curitiba, PR 80215-901 Brazil

## Abstract

**Background:**

Upon orthognathic mandibular advancement surgery the adjacent soft tissues can displace the distal bone segment and increase the load on the temporomandibular joint causing loss of its integrity. Remodeling of the condyle and temporal fossa with destruction of condylar cartilage and subchondral bone leads to postsurgical condylar resorption, with arthralgia and functional limitations. Patients with severe lesions are refractory to conservative treatments, leading to more invasive therapies that range from simple arthrocentesis to open surgery and prosthesis. Although aggressive and with a high risk for the patient, surgical invasive treatments are not always efficient in managing the degenerative lesions.

**Methods:**

We propose a regenerative medicine approach using in-vitro expanded autologous cells from nasal septum applied to the first proof-of-concept patient. After the required quality controls, the cells were injected into each joint by arthrocentesis. Results were monitored by functional assays and image analysis using computed tomography.

**Results:**

The cell injection fully reverted the condylar resorption, leading to functional and structural regeneration after 6 months. Computed tomography images showed new cortical bone formation filling the former cavity space, and a partial recovery of condylar and temporal bones. The superposition of the condyle models showed the regeneration of the bone defect, reconstructing the condyle original form.

**Conclusions:**

We propose a new treatment of condylar resorption subsequent to orthognathic surgery, presently treated only by alloplastic total joint replacement. We propose an intra-articular injection of autologous in-vitro expanded cells from the nasal septum. The proof-of-concept treatment of a selected patient that had no alternative therapeutic proposal has given promising results, reaching full regeneration of both the condylar cartilage and bone at 6 months after the therapy, which was fully maintained after 1 year. This first case is being followed by inclusion of new patients with a similar pathological profile to complete an ongoing stage I/II study.

**Trial registration:**

This clinical trial is approved by the National Commission of Ethics in Medical Research (CONEP), Brazil, CAAE 12484813.0.0000.5245, and retrospectively registered in the Brazilian National Clinical Trials Registry and in the USA Clinical Trials Registry under the Universal Trial Number (UTN) U1111–1194-6997.

**Electronic supplementary material:**

The online version of this article (10.1186/s13287-018-0806-4) contains supplementary material, which is available to authorized users.

## Background

### Clinical background

In orthognathic surgery procedures, once the mandible is advanced and fixed in its new position, the adjacent soft tissues are stretched and tend to displace the distal bone segment back to its original position. Both the amount of mandibular advancement and the degree of maxillary and mandibular rotation, associated with the rigidity of the internal fixation technique, increases the load on the temporomandibular joint (TMJ), influencing its final position and integrity [1]. The adaptive responses to this overload that remodel the condyle and the temporal fossa may be associated with transient arthralgia and functional limitations [2].

Further destruction of the condylar cartilage and of the underlying subchondral bone may extrapolate the level of adaptive tolerance leading to postsurgical condylar resorption that precipitates the development of skeletal and occlusion changes [3]. This may lead to surgical recurrence, with reduction of the mandibular ramus and clockwise rotation of the mandible, resulting in a retrognathic and hyperdivergent skeletal pattern [4], with the consequent class II malocclusion and anterior open bite. The risk is increased when similar characteristics were present before the surgery [5] and can be explained by the frequent presence of joint pathologies prior to the performance of surgical procedures in this specific group of patients [6].

Patients with severe degenerative TMJ lesions are refractory to conservative treatments that usually lead to more invasive intra-articular surgical interventions. Surgical procedures for TMJ disorders can range from simple arthrocentesis to open surgery and prosthesis. In addition to being aggressive with a high risk for the patient, a surgical invasive treatment is not always efficient in managing the degenerative TMJ lesion.

### Rationale of the regenerative medicine proposal

Regenerative medicine is a promising approach that can benefit patients with lesions that are difficult to cure, such as condylar resorption, and it should regenerate or fully replace the damaged tissues. Trauma and degenerative lesions have been the major targets of regenerative therapies in the musculoskeletal system. While bone regeneration has gained rather broad success using cells, scaffolds, and/or growth factors, cartilage regeneration has not been fully successful despite the fact that the first cell therapies were proposed for cartilage repair more than two decades ago [7]. Cartilage is avascular, with limited supplies of nutrients and without the free access to blood-born or perivascular progenitor cells, with a limited ability to promote healing and repair.

In the last decades, cell-mediated regenerative therapies have used several cell types in preclinical and clinical trials. Among them, adult multipotent stem cells and/or already differentiated cells of the mesenchymal lineages can be used safely. A large meta-analysis concerning the clinical use of such cells under different therapeutic protocols has reported no serious adverse effect related to the therapy [8], and the long-term follow-up of a large cohort of patients that has received such therapies has not found any association with an increased or altered risk of malignancies [9]. A mesenchymal cell-mediated regeneration of TMJ osteoarthritis proposal has been recently discussed as a promising option, raising possibilities of additional trophic modulation and scaffold association for cartilage and osseous compartment repair [10].

In the major joints, the mature articular hyaline cartilage layer is not covered by a perichondrium, which is substituted by only a thin membrane provided with a limited cell population. The cartilage is hence renewed by interstitial supply of new chondrocytes mobilized from the subjacent hyaline cartilage. Their replication rate is normally very low. Conversely, the TMJ condylar cartilage is covered and protected by a dense connective tissue membrane, identified as site-specific perichondrium [11]. The distal side of this membrane contains a dense collagen layer with fibroblasts only, facing the lower cavity of the TMJ. Its proximal side is overlying directly the subjacent cartilage, and it represents a potent stem cell niche that sustains a continuous proliferation of a resident population of typical mesenchymal stem cells. The molecular and functional characteristics of this niche are not well known. The external fibrous layer has a dense collagenous matrix parallel to the surface but, with increasing internal depth, the chondrogenic proliferative layer secretes a new matrix that becomes more random, progressively forming a new hyaline cartilage [12]. The classical hypertrophic chondrocyte growth, followed by mobilization of blood vessels leading to endochondral ossification, results in a continuous formation of the typical bone structure of the mandibular condyle. This permanent cell renewal originating in the TMJ perichondrium grants the condylar resistance to mechanical stress and overuse. In the TMJ, the renewal and regeneration of both cartilage and bone occur in the distal to proximal direction of the condyle. This is opposed to the growth and renewal of cells in other major joints, where the growth and renewal of bone and cartilage occur in the proximal to distal direction.

The perichondrium membrane of the condylar joint continuously produces chondroblasts, and this cell population is also required and sufficient to sustain the subjacent cartilage hypertrophic growth, endochondral ossification, and neovascularization. This means that degenerative processes such as those described in the present study, which lead to condylar arthrosis followed by condylar bone resorption, are directly a consequence of an inappropriate activity of the subperichondral progenitor cell niche [13].

The associated osteoarthritis leads to cartilage degradation and subchondral bone remodeling [13]. The regenerative approach to the therapy of such lesions should thus necessarily lead also to the control of inflammation, with the reversal of the deficient chondroblast production and the full cascade leading to endochondral bone renewal and revascularization.

We have recently shown that TMJ osteoarthritis and cartilage degradation are associated with genetic polymorphism involved in bone renewal controls, suggesting the bone-to-cartilage axis of the TMJ pathology [14]. The TMJ arthritis is classified as a “low-inflammatory arthritic condition”, but in acute condylar resorption associated with mechanical stress, such as described in the present report, both cartilage and bone tissues are heavily involved and should be equally treated. The presence of the major inflammatory mediators has recently been reported in the TMJ arthritis (reviewed in [13]), and anti-inflammatory therapy has to be one of the major concerns.

The proposed use of cells belonging to the mesenchymal lineage in TMJ osteoarthritis can directly address the question of inflammation. Mesenchymal stem/stromal cells show an intense and immediate release of mediators that control inflammation. This can involve either the production and secretion of cytokines, the release of regulatory epigenetic factors in exosomes, or the induction of resident macrophages expressing the M1 proinflammatory phenotype towards the M2 anti-inflammatory phenotype [15, 16]. Mesenchymal cells can thus simultaneously control the inflammatory environment and provide the progenitor cell population required for the repair and regeneration of chondrogenesis in the condylar apical perichondrium, promoting the subsequent endochondral ossification and bone repair.

The final question of the proposal was the choice of the cells to be used in the TMJ regeneration. The mesenchymal cell lineage includes a broad set of substrate-adherent cells of different origins and capacities, able to proliferate and differentiate into cells with specific functions [17]. According to the International Society for Cellular Therapy, the minimal characterization of mesenchymal stem/stromal cells requires their phenotyping through identification of the surface membrane markers, which should be positive for CD105, CD73, and CD90, and negative for CD34, CD45, CD14 or CD11b, CD79a or CD19, and HLA-DR [18]. However, when mesenchymal cells are maintained and expanded in vitro, expression of surface markers can be modulated, and a considerable variability in expression of several markers can be observed among mesenchymal stem cells of different tissue origins [19]. The capacity to differentiate, when appropriately stimulated, in at least a few differentiated cell types such as osteoblasts and chondroblasts is also required in the same definition proposal [18].

Cells of the mesenchymal lineage derive from mesoderm or from ectomesoderm of the neural crest [20]. Cells of cranial bones and cartilage, including mandible and TMJ, are derived from the neural crest cell pool. Clonogenic studies of the neural crest-derived stem cells have shown that a vast majority of them can yield all the craniofacial skeleton cells in addition to the neural cells including glia and melanocytes. Skeletogenic progenitors give also rise to myofibroblasts belonging to the phenotype of pericytes and resident progenitor cells of the mesenchymal lineages [21].

To remain within the cell lineages derived from the neural crest progenitors, in the present study we have used autologous cells derived from the nasal septum. These cells have a high proliferative activity and can be easily harvested. Previous studies have shown that adult human neural crest-derived cells implanted in animal cartilage tissue of mesodermal origin can be reprogrammed and contribute to cartilage repair [22]. A subsequent study has shown similar results in humans; radiological assessments indicated tissue repair, potentially mediated directly or indirectly by the nasal septum-derived cells [23].

The nasal septum structure is similar to the condylar cartilage. It is covered by a perichondrium with a dense external fibrous layer and a subjacent layer containing proliferating progenitor cells of the hyaline cartilage. In large trunk fibrocartilaginous tissues, this layer is clearly recognized and distinguished as the cambium layer. In the nasal septum, the border between the two layers is not clear, similar to that observed in the condylar perichondrium. In a previous study of human nasal septum-derived cells, we have harvested the layers overlying the hyaline cartilage by a brief collagenase digestion of perichondrium, and we studied the mesenchymal progenitors in this cell population [24]. We showed that cartilage progenitors were present in this perichondrium and that they were highly proliferative and able to differentiate into chondrocytes and osteoblasts, but not into adipocytes. These cells were expressing the Sox9-gene, recognized to be a marker of chondrocyte and osteoblast progenitors, required for both endochondral and intramembranous ossification [25]. In view of the observations that hyaline cartilage chondrocytes plated onto a solid substrate can also dedifferentiate and proliferate, maintaining the prochondrogenic phenotype, we harvested and processed a full thickness of the septum to be used in the therapies proposed here. The obtained cell populations have been shown to be highly proliferative and able to promote regeneration of both cartilage and bone.

## Methods

The present study has been approved by the Petrópolis Faculty of Medicine Committee of Ethics in Research (CEP-FMP) and by the National Commission of Ethics in Medical Research (CONEP), CAAE 12484813.0.0000.5245. The study was retrospectively registered in the Brazilian National Clinical Trials Registry, and in the USA Clinical Trials Registry, under the Universal Trial Number (UTN) U1111–1194-6997.

The first patient, a 27-year-old male, was included after signing a consent form. The clinical profile and evolution of the patient are described in the Results section. After an indication for the regenerative medicine approach and his inclusion in the present study, the patient was submitted to a nasal cartilage biopsy for isolation and preparation of the nasal septum-derived chondrocytes. This procedure was performed during surgical intervention under general anesthesia. On the same day, 20 mL of the patient’s blood was collected to prepare autologous serum.

**Fig. 1:**
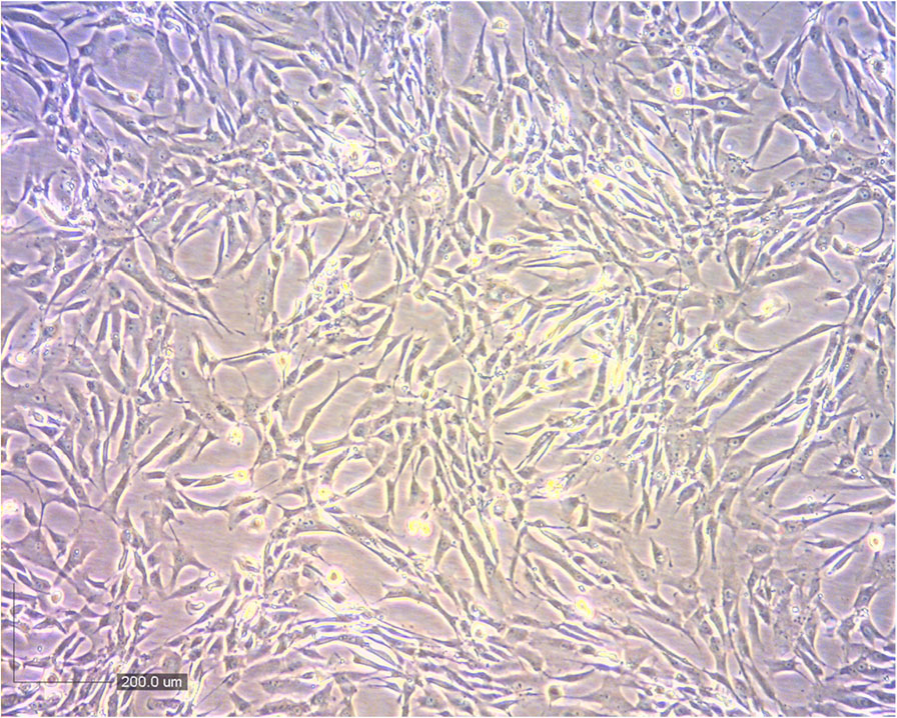
The in-vitro cell morphology of the mesenchymal cells obtained from the nasal septum, plated in culture flasks and cultivated in a humidified atmosphere containing 5% CO_2_ at 37 °C

For further processing, the biological samples were transported under temperature-controlled conditions (2–8 °C) to the Cellular Technology Center of the Pontifical Catholic University of Paraná, Curitiba, PR, Brazil. The cartilage was minced into small fragments and digested with 0.1% (*w/v*) collagenase solution (Collagenase Type Ι, Sigma) at 37 °C for 3 h under gentle agitation. The obtained cells were plated in culture flasks (4 × 10^4^ cells/cm^2^) in Dulbecco’s modified Eagle’s medium (DMEM; Sigma-Aldrich) supplemented with 20% fetal bovine serum (FBS), 10 mg/mL ciprofloxacin, 2.5 μg/mL amphotericin, and 2 mM l-glutamine (Sigma-Aldrich), and cultivated in a humidified atmosphere containing 5% CO_2_ at 37 °C. The *in-vitro* cell morphology is shown in Fig. 1. Cells were cultured for 21 days up to passage 2 (P2) and were cryopreserved. One week prior to injection, cells were thawed and cultivated for one more passage (P3) and 2 days prior to injection FBS was replaced by autologous serum. On cell preparation day, samples were collected for quality control. Cells were suspended in phosphate-buffered saline (PBS), supplemented with 25% *v*/v commercial injectable solution containing 5 mg/mL sodium hyaluronate (Osteonil®, TRB Pharma) and with 5% autologous serum. A cell suspension with 10 × 10^6^ cells in 1 mL was placed in each the two cryogenic tubes. The final volume injected in each TMJ was 1 mL containing 10^7^ cells (time 1 (T1)) by arthrocentesis [26].

Quality control of the cell suspension sterility was evaluated by tests to detect bacteria and fungi (Bact/Alert 3D, Biomerieux), endotoxins (Endosafe™ PTS, Charles River), and mycoplasma (KIT MycoAlert™ PLUS Mycoplasma Detection, Lonza). Cell viability was performed by flow cytometry using the vital dye 7-aminoactinomycin D (7-AAD; BD #559925) to determine the percentage of viable cells, and Annexin V protein (BD #51-65,875X) to determine the percentage of cells in apoptosis. Cytogenetic analysis was performed by the GTG-banding method.

The cells were characterized before the clinical application by flow cytometry, following the expression of the surface makers (Additional file 1). Cells were washed with PBS containing 2% FBS and incubated with the monoclonal antibodies: fluorescein isothiocyanate (FITC)-labeled CD14 (BD #555397), CD45 (BD #555482), CD19 (BD #555412), CD44 (BD #555478); phycoerythrin (PE)-labeled CD73 (BD #550257), CD90 (BD #555596), CD166 (BD #559263), CD151 (BD #556057), CD49C (BD #556025); PerCP-labeled HLA-DR (BD #551375); and APC-labeled CD34 (BD #555824), CD105 (BD #562408), CD29 (BD #559883), all purchased from BD (Pharmingen). At least 100,000 events were acquired on a BD FACSCalibur™ flow cytometer (BD Biosciences) and the data were analyzed using FlowJo 10 (TreeStar) software. We also performed assays of potential differentiation induction of nasal septum-derived mesenchymal cells (the fourth passage) to control the quality of injected cells (Additional file 2). When maintained under standard culture conditions, as described before, the viability of the cells was 95.79%. All the sterility tests were negative and only nonclonal chromosomal abnormalities were observed. The cells displayed the typical fibroblastoid morphology, characteristic of mesenchymal cells. In the fourth passage, the cells were seeded on glass coverslips in 24-well plates (Additional file 2: A–D). Cells were maintained under standard culture conditions (Additional file 2: A and C). In order to monitor their potential adipogenic and osteogenic differentiation, they were incubated with the corresponding commercial differentiation media (Lonza, Basel, Switzerland) for 21 days (Additional file 2: B and D). Cells were fixed and stained with Oil Red O to detect triglyceride accumulation and confirm adipogenesis (Additional file 2: B), or with Alizarin Red S to detect calcium deposition and osteogenic differentiation (Additional file 2: D). Alternatively, in order to form nodules (Additional file 2: E and F), cells were centrifuged at 300 g for 10 min to form a pellet. Without disturbing the pellet, cells were cultured for 21 days in standard medium or in the commercial chondrogenic differentiation medium (Lonza, Basel, Switzerland). The nodules were fixed in 10% formaldehyde for 1 h at room temperature, dehydrated in serial ethanol dilutions, and embedded in paraffin blocks (Additional file 2: E and F). Paraffin sections (4 μm thick) were stained for histology with Toluidine Blue solution (Sigma-Aldrich, St. Louis, USA) to demonstrate intracellular mucopolysaccharides. No differentiation in adipogenic and osteogenic lineages was observed after 21 days of culture. In chondrogenic differentiation assays, high-density micromass cell cultures generated cellular nodules that produced large amounts of cartilage-related extracellular matrix molecules. Paraffin sections of the aggregates stained with toluidine blue showed a cartilaginous extracellular matrix stained in purple (metachromasia), showing the highly sulfated proteoglycans of cartilage matrices, while undifferentiated or fibrous tissue stained in blue. In conclusion, mesenchymal cells derived from the nasal septum maintained spontaneously the chondrocyte phenotype. When exposed to culture conditions that can induce in-vitro adipogenic or osteogenic differentiation in bone marrow-derived mesenchymal stem/stromal cells, they did not respond to this induction and maintained only their chondrogenic phenotype.

Since the cell transport could take up to 48 h and cell injection included the addition of hyaluronic acid, we monitored the cell viability under these conditions. Two groups of cells were prepared in PBS supplemented with 5% autologous serum and 25% hyaluronic acid. The first group had the hyaluronic acid added immediately and maintained during the predicted transport times and conditions. The second group had the hyaluronic acid added only after 24 or 48 h that corresponded to the moment of application into the TMJ. We monitored the percentage of cells that entered apoptosis or suffered necrosis by cell suspension incubation with Annexin V (BD #51-65,875X) that stains apoptotic cells, and with 7-AAD (BD #559925) that detects necrotic cells. The relative content of apoptotic and necrotic cells in the suspension were monitored by flow cytometry and the percentage of viable cells was expressed in relation to the total number of cells (1 × 10^7^) to be implanted in each TMJ. We observed viability of 98.22% of cells immediately after the cell suspension preparation. The percentage of viable cells decreased with time in both groups (that either received hyaluronic acid from the beginning of suspension preparation or that received hyaluronic acid at the time corresponding to cell injection). In the former group, viable cells reached 94.04% and 78.6% at 24 h and 48 h, respectively, and in the latter group reached 88.4% and 88.9%, respectively. The differences between the groups were not statistically significant.

Clinical follow-up was performed at 7 and 15 days and at 1, 3, and 6 months after the treatment. The results were considered effective when a decrease in the intensity and severity of pain was experienced according to the Diagnostic Criteria for Temporomandibular Disorders (DC/TMD) questionnaire [27], and cartilage regeneration was demonstrated by three-dimensional computed tomography (CT) image superposition after a 6-month follow-up (time 2 (T2)).

The images were acquired in a Cone Beam CT “I-Cat Classic” (Imaging Science International®) using the extended height protocol: field of view 16 × 22 cm, scan time 40 s, and voxel size 0.4 mm. The acquisition protocol was the same at T1 and T2 to avoid differences in image resolution. The acquired images were saved in DICOM file format that can construct three-dimensional volumetric files of the regions of interest for superposition, a process known as segmentation. Therefore, the ITK-SNAP 3.6 software (www.itksnap.org) was applied. Three-dimensional surface mesh models of the right and left mandibular condyles at T1 and T2 were constructed by outlining the cortical boundaries of the condylar region using semiautomatic discrimination procedures that also allows manual editing. All condylar models were then cropped to a more defined region of interest consisting of only the condyle and a mandibular ramus portion. After the segmentation, the next step of image analysis consisted of registering the scans and their respective three-dimensional volumetric models in a common coordinate system using a target region as the reference. This was done with 3D slicer 3.1 software (www.3dslicer.com); this procedure allows for different types of registrations used for reference, as landmarks, surface models, or voxel gray intensity. In the present study we have chosen it for reference of registration, the option of the surface models.

After the registration of the scans, the visual analysis of three-dimensional morphological and volumetric changes could be performed. We used the 3D Slicer software with the extensions modules ModelToModelDistance (http://www.nitrc.org/projects/meshmetric3d) and ShapePopulationViewer 1.3.2 (https://www.nitrc.org/projects/shapepopviewer), which allow the measurement of the distance between two three-dimensional models and to visualize and compare the surfaces at the same time.

The ModeltoModelDistance and the ShapePopulationViewer modules illustrate the difference between distances of three-dimensional models through color variation. To assess the results in left and right condyles after the injection procedure, the initial model at T1 and final model at T2 were considered. The color difference has a millimeter distance ranging from 0 to 2.5 mm, defined by the sequence of colors green (0), yellow (1.75 mm), and red (2.5 mm). These color schemes facilitate visualization of morphological and volumetric changes in the models, where green areas show that there was no difference between the initial and final models, a yellow color identifies moderate changes, and a red color identifies marked differences between models.

## Results

The viability of the cells after processing was 95.79%; all the sterility tests were negative and only nonclonal chromosomal abnormalities were observed.

The studied cells showed specific surface markers as follows: high expression of CD73 (87.2%), CD29 (95.5%), and CD166 (83.7%); moderate expression of CD105 (65.1%); and very low expression of CD14 (0.92%), CD34 (0.35%), CD45 (0.47%), CD19 (0.61%), and HLA-DR (6.02%). This indicated their mesenchymal characteristics. They also highly expressed CD49c (95.1%), CD151 (96.8%), and CD44 (82.5%), indicating their chondrogenic profile (Additional file 1).

**Fig. 2:**
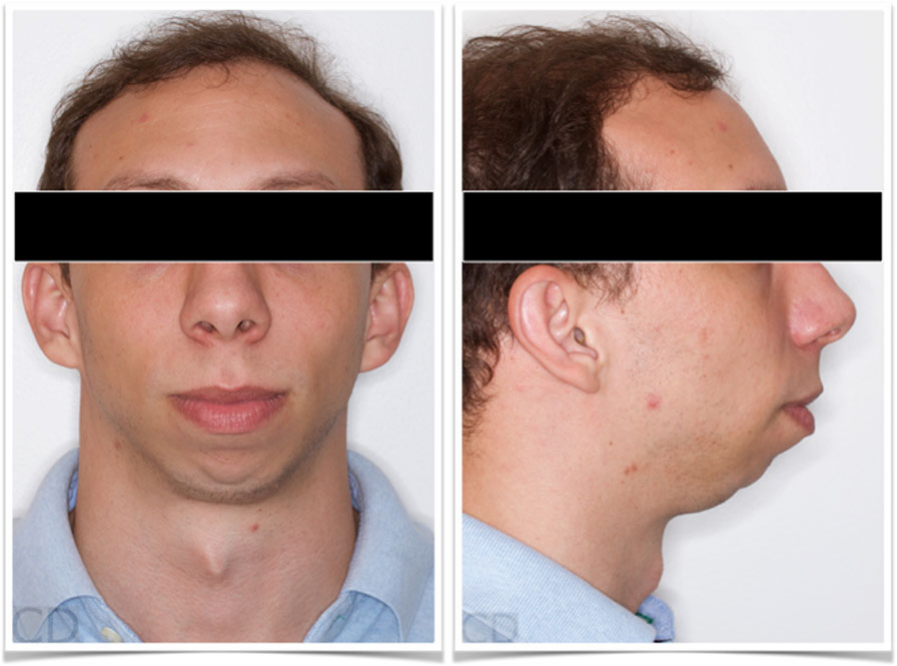
Extra-oral photos of a 27-year-old male patient with a severe skeletal class II and hyperdivergent dentofacial deformity

**Fig. 3:**
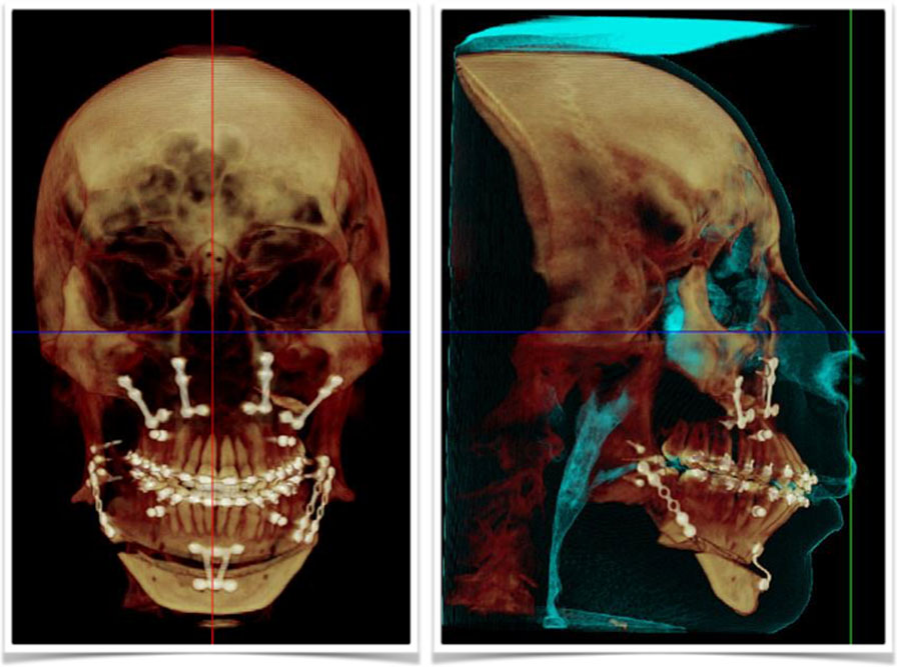
Le Fort I maxillary surgery combined with bilateral mandibular sagittal split and mentoplasty procedure were performed with rigid fixation

**Fig. 4:**
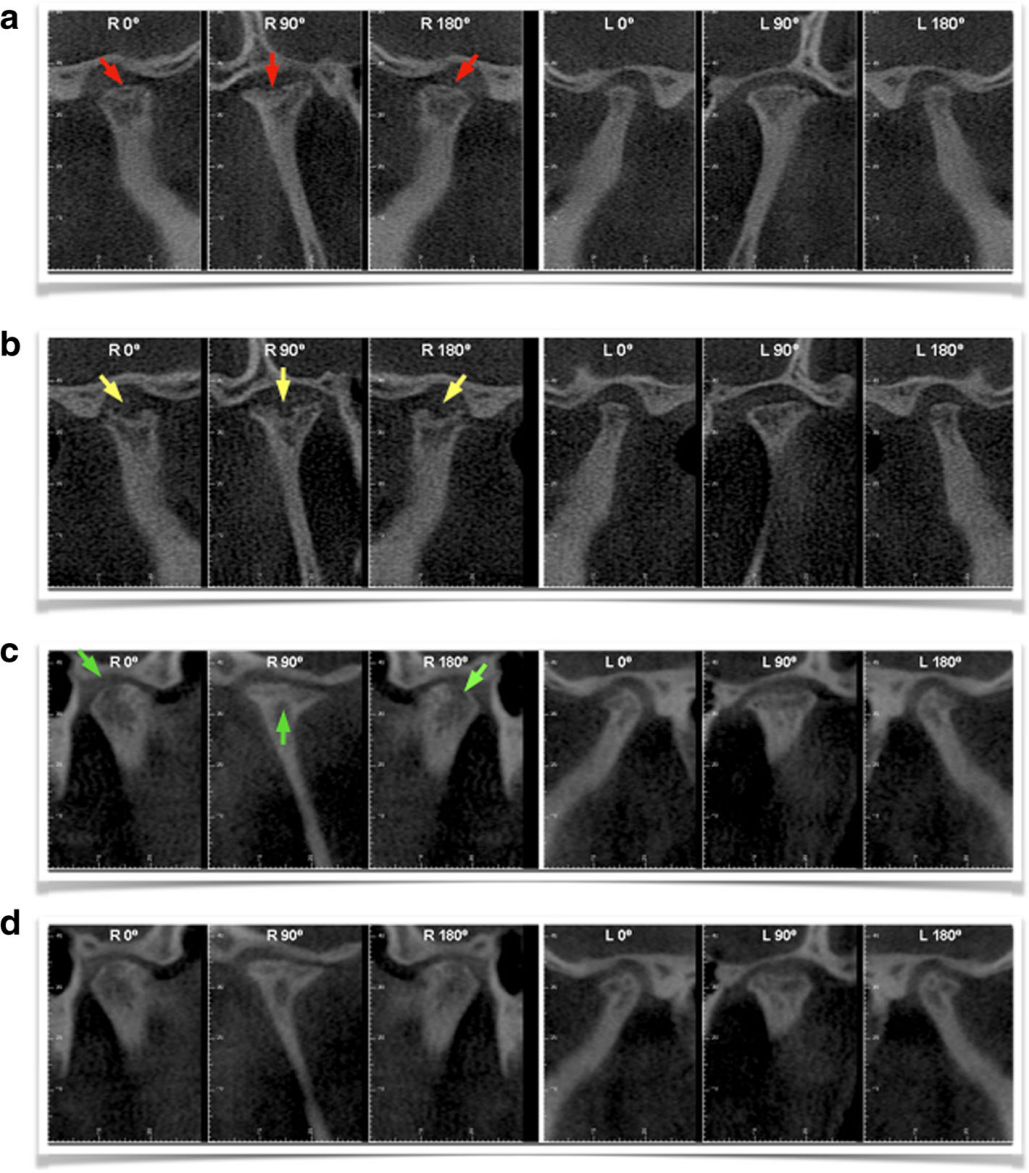
Right and left TMJ CT images from the patient at different follow-up times: external lateral view (0°), frontal view (90°), and internal lateral view 180°) (**a**) CT images obtained before first orthognathic surgery. Note the presence of degenerative changes in both TMJs, being more severe in the right one (red arrows). (**b**) CT images obtained 6 months after first orthognathic surgery. Note worsening of degenerative changes in the right side, with a bone cavity formation (yellow arrows). (**c**) CT images obtained 6 months after cell therapy injection. Note the new cortical and subcortical bone formation (green arrows). (**d**) CT images obtained 1 year after the injection of the cellular therapy, demonstrating the maintenance of the gains obtained during the former follow-up period

The patient included in the present study had a severe skeletal class II hyperdivergent dentofacial deformity due to mandibular micrognathia, with an indication of orthognathic surgery for its correction (Fig. 2). He had been submitted to a maxillo-mandibular advancement with counter-clockwise rotation of the entire complex and mentoplasty procedure (Fig. 3). The CT images obtained before the surgery (T0) already showed degenerative changes in both TMJs, being more severe in the right one (Fig. 4a).

The patient was kept under the use of intermaxillary orthodontic elastics for maintenance of the achieved occlusion due to a severe tendency for postsurgical mandibular relapse which eventually led to maxillary loading and pseudoarthrosis. A new surgical procedure was required to fix the maxilla and this was performed 6 months after the first one.

At the moment of the cell therapy injection, the patient presented with a relapse of the former orthognathic surgical procedure, compromising its esthetical and functional results, and presented a marked skeletal retrognathic pattern.

The new CT images showed a progression of degenerative changes of both TMJs, with intense condyle resorption especially in the superior area of the right one showing an image that seemed to be a bone cavity (Fig. 4b).

The patient presented a good clinical evolution during the 6-month period post-transplantation. At his inclusion into the study, the Von Korff index for chronic pain severity showed a low pain intensity (< 50) without disability (grade I). The score of mandibular functional limitation was moderate, with maximum passive mouth opening without pain of 19 mm and maximum active mouth opening of 23 mm. During the clinical examination, we identified sounds recognized as crepitus in both joints, with intermittent locking during routine mouth opening. The joint pain was registered during palpation only on the left TMJ.

The first 7 days of clinical evaluation showed mild pain on the injection site which was fully controlled by a pain-killer prescription in the postsurgery period; this was the only adverse effect observed in this first follow-up week. There was no need for medications controlling arthralgia, and there was no pain at 15 days postsurgery follow-up visits. The maximum assisted mouth opening improved, yet it was limited to an amplitude of 30 mm. The patient reported a moderate headache probably not related to the injection procedure that resolved spontaneously.

After 1 month of follow-up, the patient persisted without any temporomandibular or other facial pain, and there was no pain at rest or chewing. The patient’s subjective evaluation of treatment efficacy and tolerability was excellent. Von Korff index of chronic pain severity was zero as the patient experienced no temporomandibular pain at all. The functional limitation was scored by the patient as moderate; once the maximum assisted mouth opening had a mild decrease to 27 mm, probably due to the functional restriction related to the pseudoarthrosis recovery process. It was not possible to clinically identify any joint sounds or pain during TMJ palpation. The only new adverse effect reported were three consecutive episodes of night sweats, probably not related to the injection procedure.

Three months after the cell injection, the patient persisted without any pain medications. The subjective evaluation of his own masticatory efficiency was now satisfactory, without any temporomandibular pain during rest or chewing. The limitation of functional movements was only mild because of pseudoarthrosis resolution and was potentially improving following the beginning of physical therapy. This involved a therapist for 60 min once a week for 10 weeks. The technique comprised mobilizing the TMJs with traction and translation movements in all directions. After the first four appointments, the patient was instructed to execute all exercises at home, four sets of 2 min in front of a mirror.

The patient’s subjective evaluation of treatment efficacy and tolerability was still classified as excellent. A clicking sound was perceived at the left joint during mandibular opening movement without any arthralgia on palpation during clinical examination. The Von Korff scale persisted at a score of zero. The functional mandibular limitation was scored as mild at this time with maximum, unassisted and assisted, mandibular opening of 30 mm and 31 mm, respectively. No adverse effects of any kind were reported.

At the 6-month follow-up visit, all the subjective and objective parameters remained at the same levels. The masticatory efficacy improved to a score classified as good, and mandibular opening parameters improved by 1 mm probably by the removal of chewing restrictions related to the pseudoarthrosis and the physical therapy intervention. Again, no adverse side effects were reported. At this time, new laboratory tests and CT images of the TMJ were obtained.

At the 1-year follow-up visit, the patient’s subjective self-reported treatment efficacy was excellent, with no complaints of pain at rest or during mandibular function. The masticatory efficacy improved to the score of excellent. Mandibular assisted opening reached 36 mm. Again, no adverse side effects were reported. At this time, new laboratory tests and CT images of the TMJ were obtained demonstrating the maintenance of the gains achieved during the former follow-up period.

Laboratory tests in the acute post-transplant phase demonstrated the absence of an acute inflammatory reaction: C-reactive protein was within normal levels, with unchanged blood count, and glucose, urea, creatinine, sodium, and potassium at normal levels. There was no change in weight over the 6-month period. The patient did not report pain, fever, or any other clinical complication.

**Fig. 5:**
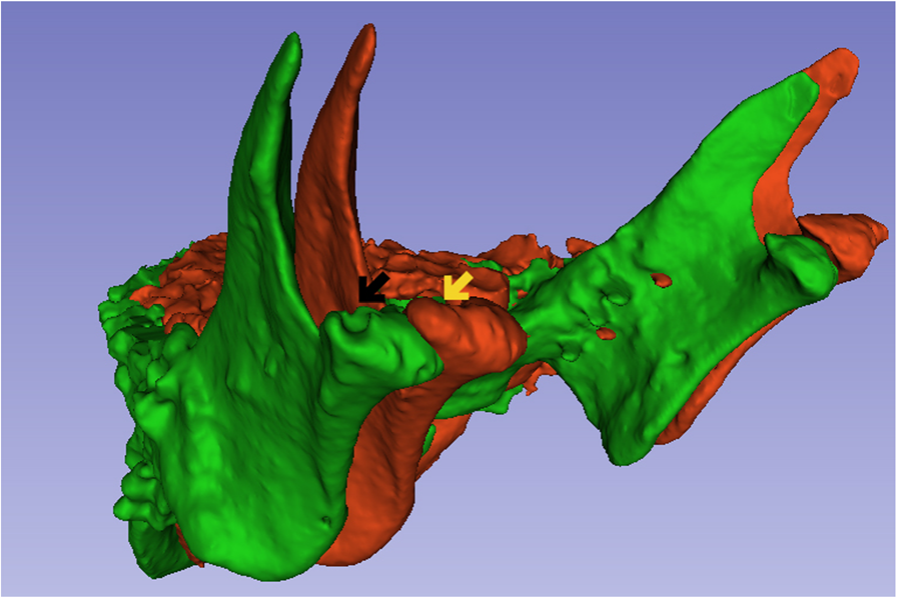
CT image three-dimensional reconstruction, being green before and red after the cell therapy injection. Note the filling of the bony defect in the right TMJ (black and yellow arrows)

**Fig. 6:**
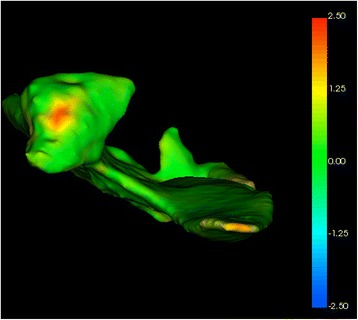
CT three-dimensional image color superposition scheme for visualization of morphological and volumetric changes. Green areas show no difference between the initial and final models; yellow color identifies moderate changes (up to 1.5 mm); red color identifies marked differences (up to 2.5 mm) between the models before and after the experimental therapeutic procedure

The CT analysis revealed evidence of a regenerative process following the autologous chondrocyte transplantation. The two-dimensional reconstructed CT image analysis showed a visible new cortical and subcortical bone formation on the right joint, filling the cavity space created by the condylar resorption process, with a decrease in the articular space and a partial recovery of condylar and temporal bone process anatomy (Fig. 4c and d). From T1 to T2, CT image three-dimensional reconstruction (Fig. 5) and the right condyle model superposition (Fig. 6) showed the regenerative process filling the cavity defect on the right side, partially reconstructing the original form of the condyle.

## Discussion

Condylar resorption following the surgical correction of mandibular retrusion was first described in 1985 [28]. The first signs are usually detected up to 6 months after the orthognathic surgery, as observed in the present case report, and the process may extend for up to 2 years. The extension of this period is related to the magnitude of the long-term skeletal recurrence [29]. Patients affected by this process may be asymptomatic. The reported pain intensity does not necessarily correlate with the severity of the observed degeneration, as seen in the case reported here in which the patient presented low pain intensity without disability at the time of inclusion in the study.

This progressive and slow surgical relapse was, until the present time, considered irreversible. Clinical treatment proposals are limited, ranging from palliative care to invasive surgical interventions. A systematic review evaluating current treatments for this dentoskeletal deformity due to condylar resorption and their outcomes demonstrated that less than 10% of all cases received a conservative treatment [30]. Orthognathic surgery was the most commonly used option, sometimes combined with open joint surgery. Temporomandibular total joint prostheses were used in 10% of all cases, with stability observed in almost all the patients. Consequently, alloplastic total joint replacement is now considered as the gold standard for reconstruction of the irreparably damaged adult TMJ [31].

A statistical projection of the number of alloplastic TMJ total replacements to be performed in the United States through the year 2030 indicates an increase of nearly 60%, reaching up to 900 prostheses per year [32] with high total costs ranging from U$12,000 to U$15,000 for a bilateral replacement [33]. No projection is available on the possible long-term cost of cell therapy to be used for TMJ degeneration, but a comparable economic evaluation of autologous chondrocyte implantation in the knee has been recently published [34]. This reports that autologous chondrocyte transplantation for symptomatic articular cartilage defects of the knee has been more cost-effective across a broad range of scenarios, even when compared with a simple microfracture surgical procedure without the use of prosthesis.

The current TMJ total joint prostheses register now reaches up to 20 years follow-up with satisfactory outcomes. It remains to be seen whether the results can be maintained over 20 years, since the average age for TMJ replacement is close to 40 years. The metal or high molecular weight polyethylene joints seem to have similar outcomes. They are similar to constructs used in total knee replacements which are used well beyond 10 years and start to fail at 20 years due to wear debris. No case of wear debris has yet been reported following TMJ replacement with these prostheses, but it is too early to reach final conclusions [35].

The failure of TMJ prostheses implants that were eventually explanted and replaced has been reported in a short-term 2-year follow-up to reach 5% [36]. The reasons for implant removal and replacement include heterotopic bone formation, infection, and/or loose hardware. The most worrying complication is infection, which may affect up to 4.5% of patients [37].

Autologous chondrocyte implantations have been studied since 1987, and the potential adverse effects are relatively well known. The procedure has evolved over time and the failure rates show a reduction to less than 1% in all arthroscopic second-generation autologous chondrocyte implants, while unplanned reoperation rates are close to 1.4% [38].

Biomechanical issues are one of the major concerns of TMJ total joint replacements. Those reported for alloplastic reconstructions of TMJ were evaluated and compared with TMJ of healthy controls. Quantitatively, mandibular movements of artificial joints during opening, protrusion, and laterotrusion were all significantly shorter than those of controls. A significantly restricted mandibular range of motion in replaced joints was also observed clinically. Fifty-three percent of patients suffered from chronic pain at rest, and 67% reported reduced chewing function. Nonetheless, patients declared a high level of satisfaction with the replacement [39].

The timing and the long-term evolution of regenerative cell therapy in articular cartilage is of interest when comparing them to prostheses. Studies that monitored the effect of the chondrocyte implant into knee cartilage lesions observed a delay in reaching maximal functional improvement as compared with other interventions, but the overall long-term durability was potentially longer in cell therapy [34]. Up to 80% patients improved by 15 months, and this improvement was sustained for 9 to 16 years [40]. Moreover, the clinical improvement was maintained over time as well as the patient’s satisfaction with the cellular implant. Ten to 20 years after the implantation, 74% of patients reported their status as better or the same as the previous years, and 92% were satisfied and would have the cell implant again [41]. This late improvement may be a consequence of re-establishment of the tissue regeneration processes in the patient. The quality of regenerated cartilage tissue after chondrocyte implantation was reported to be better than that obtained by microfractures [34], and this cell-based therapy may have a higher potential to regenerate hyaline-like tissues in the treatment of large sized and full-thickness cartilage defects [42].

The patient described here received an autologous chondrocyte-derived cell-based therapy for condylar resorption in a proof-of-concept context for this newly proposed therapy for TMJ. His state has improved throughout the 1-year follow-up. The cartilage repair was followed by a full regeneration of the condylar bone resorption. This was proposed and expected in view of the normal bone renewal from the mesenchymal cell niche of the intra-articular perichondrium in this joint. The ongoing study in a series of patients should give information on the reproducibility and extension of this therapeutic proposal in similar clinical contexts.

## Conclusions

We have elaborated and described here a new proposal for the treatment of condylar resorption subsequent to orthognathic surgery, which receives at present only an alloplastic total joint replacement for reconstruction of the irreparably damaged adult TMJ. We propose an intra-articular injection of autologous in-vitro expanded cells obtained from the nasal septum. The proof-of-concept treatment of a selected patient that had no alternative therapeutic proposal has shown promising results with a full regeneration of both the condylar cartilage and bone attained 6 months after therapy. The result has remained stable after 1 year of follow-up. This first case is being followed by inclusion of new patients with a similar pathological profile in order to complete a stage I/II study.

## Additional files

Additional file 1: *Immunophenotypic characterization of the surface CD markers by flow cytometry.* The blue histograms indicate the percentage of the positive expression for each antibody while the red histograms indicate the isotype control. (A) Surface markers for the characterization of mesenchymal stem cells. (B) Surface markers indicating the chondrogenic profile. The cells were characterized before their clinical application. (PDF 351 kb)

Additional file 2: Mesenchymal cells derived from the nasal septum maintained spontaneously the chondrocyte phenotype. The figure shows assays of potential differentiation induction of nasal septum-derived mesenchymal cells. Cells represented in (A) and (C) were maintained under standard culture conditions, and Cells represented in (B) and (D) correspond to the adipogenic and osteogenic differentiation test, respectively. Cells were fixed and stained with Oil Red O to detect triglyceride accumulation (B), or with Alizarin Red S to detect calcium deposition (D). Alternatively, in order to form nodules (E and F), cells were induced to chondrogenic lineage. Paraffin sections of the aggregates stained with toluidine blue showed a cartilaginous extracellular matrix stained in purple (metachromasia), showing the highly sulfated proteoglycans of cartilage matrices, while undifferentiated or fibrous tissue stained in blue. No differentiation in adipogenic and osteogenic lineages was observed after 21 days of culture. In conclusion, mesenchymal cells derived from the nasal septum maintained spontaneously the chondrocyte phenotype. (PDF 952 kb)

## Abbreviations

7-AAD: 7-Aminoactinomycin
CT: Computed tomography
FBS: Fetal bovine serum
FITC: Fluorescein isothiocyanate
PBS: Phosphate-buffered saline
TMJ: Temporomandibular joint
