# Supplementary figures and images for: Temporomandibular joint regeneration: proposal of a novel treatment for condylar resorption after orthognathic surgery using transplantation of autologous nasal septum chondrocytes, and the first human case report
Source: Stem Cell Res Ther. 2018 Apr 7;9:94. doi: 10.1186/s13287-018-0806-4 (PMC5889586; doi:10.1186/s13287-018-0806-4)

**A**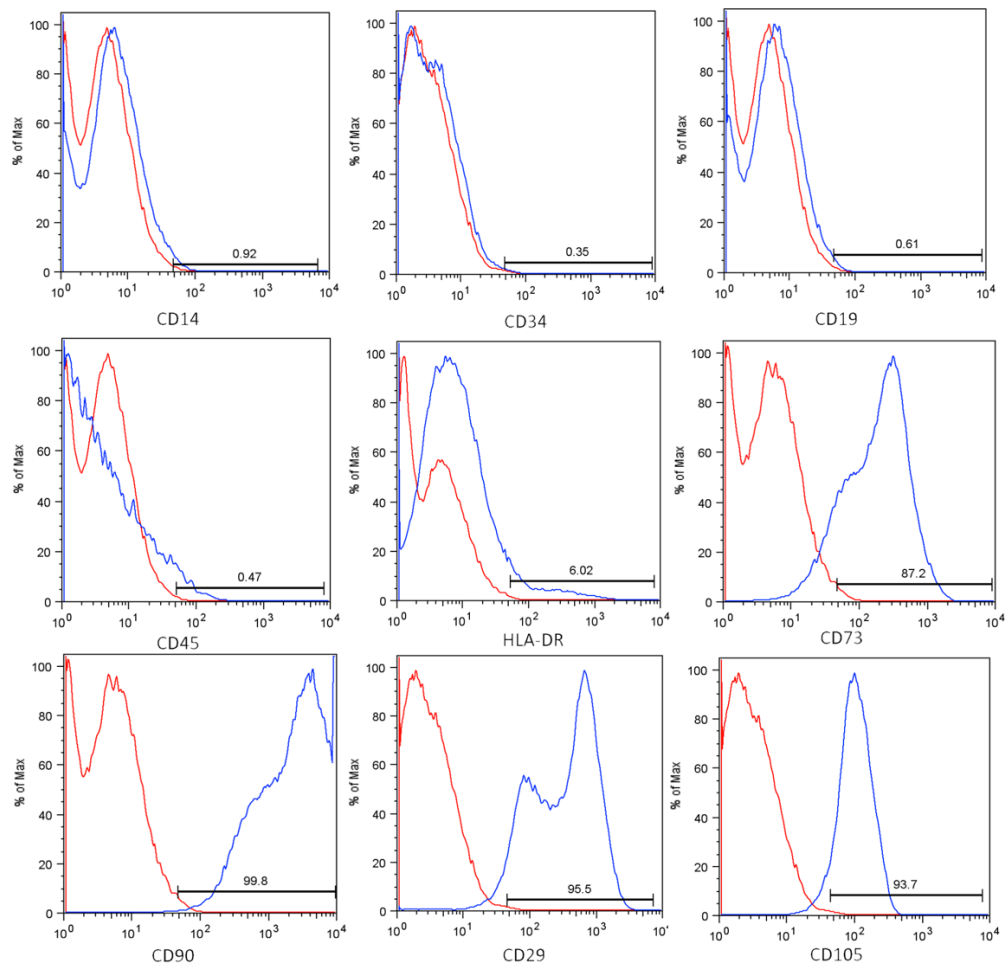**B**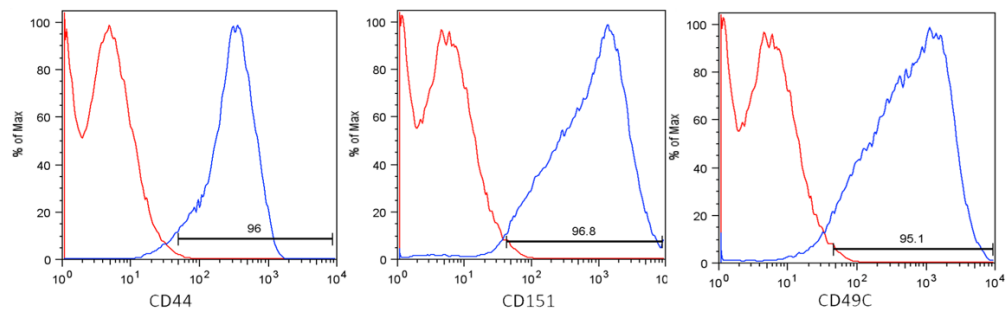

Supplement: Supplementary file 1 — Immunophenotypic characterization of the surface CD markers by flow cytometry. The blue histograms indicate the percentage of the positive expression for each antibody while the red histograms indicate the isotype control. (A) Surface markers for the characterization of mesenchymal stem cells. (B) Surface markers indicating the chondrogenic profile. The cells were characterized before their clinical application. (PDF 351 kb) [file 13287_2018_806_MOESM1_ESM.pdf]

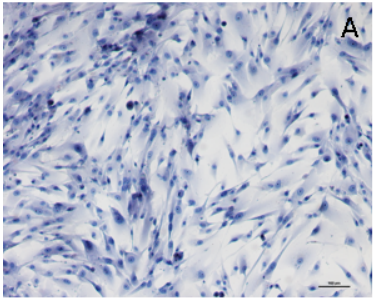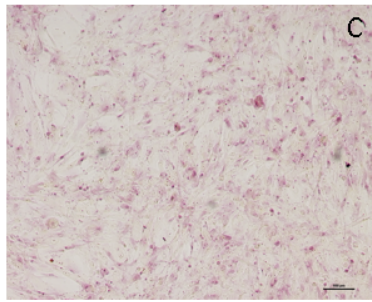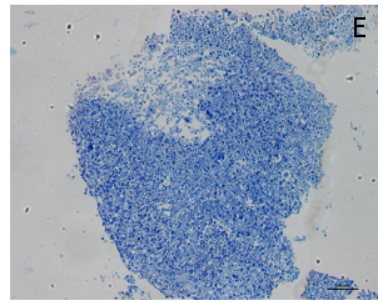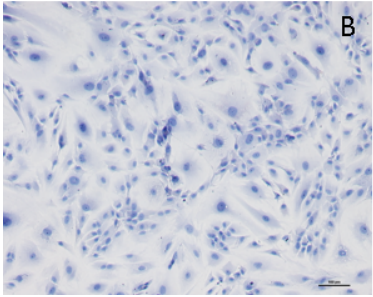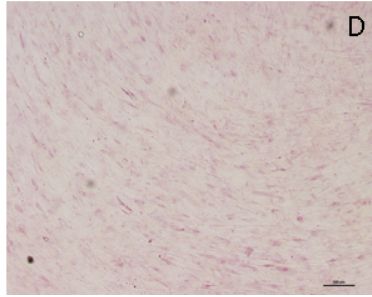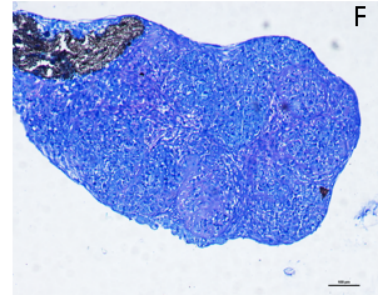

Supplement: Supplementary file 2 — Mesenchymal cells derived from the nasal septum maintained spontaneously the chondrocyte phenotype. The figure shows assays of potential differentiation induction of nasal septum-derived mesenchymal cells. Cells represented in (A) and (C) were maintained under standard culture conditions, and Cells represented in (B) and (D) correspond to the adipogenic and osteogenic differentiation test, respectively. Cells were fixed and stained with Oil Red O to detect triglyceride accumulation (B), or with Alizarin Red S to detect calcium deposition (D). Alternatively, in order to form nodules (E and F), cells were induced to chondrogenic lineage. Paraffin sections of the aggregates stained with toluidine blue showed a cartilaginous extracellular matrix stained in purple (metachromasia), showing the highly sulfated proteoglycans of cartilage matrices, while undifferentiated or fibrous tissue stained in blue. No differentiation in adipogenic and osteogenic lineages was observed after 21 days of culture. In conclusion, mesenchymal cells derived from the nasal septum maintained spontaneously the chondrocyte phenotype. (PDF 952 kb) [file 13287_2018_806_MOESM2_ESM.pdf]
